# Validity and reliability of the Arabic version of the revised illness perception questionnaire for patients with hypertension

**DOI:** 10.3389/fpubh.2022.874722

**Published:** 2022-09-28

**Authors:** Sameer Al-Ghamdi, Alhaytham Mohammed Al Muaddi, Nawaf Ali Alqahtani, Tamim Yahya Alhasoon, Abdulaziz Abdullah Basalem, Abdulrahman Abdullah Altamimi

**Affiliations:** ^1^Department of Family and Community Medicine, College of Medicine, Prince Sattam Bin Abdulaziz University, Al-Kharj, Saudi Arabia; ^2^Undergraduate Medical Student, Department of Family and Community Medicine, College of Medicine, Prince Sattam Bin Abdulaziz University, Al-Kharj, Saudi Arabia

**Keywords:** validity, reliability, Arabic version, revised illness perception questionnaire, hypertension

## Abstract

**Background:**

Hypertension is one of the leading causes of morbidity and mortality in Saudi Arabia affecting 31.4% of the population. The Illness Perception Questionnaire-Revised (IPQ-R) is a validated and reliable tool for assessing the perception of hypertension among patients. This cross-sectional study aimed to translate the Revised Illness Perception Questionnaire (IPQ-R) into Arabic and validate it among Arabic patients with hypertension from the outpatient departments of the Prince Sattam University Hospital and King Khalid Hospital (KKH) in Al-Kharj City in the Kingdom of Saudi Arabia.

**Methods:**

A bilingual panel of doctors and medical translators was assembled to translate the IPQ-R into Arabic. The questionnaire was administered to 100 adult Arabic speaking patients with clinically diagnosed primary hypertension. Patients with secondary hypertension or complications of hypertension were excluded from the study.

**Results:**

Fifty-seven patients (57%) were male and sixty-five (65%) were older than 40 years. Headache was the most common symptom of hypertension reported by 65% of the participants. The internal consistency of the questionnaire excluding the domain of ‘Disease Identity' was 0.76 indicating satisfactory consistency. There were weak to moderate positive linear correlations (r = 0.003–0.561) between the domains of IPQ–R suggesting a reasonable discriminant validity among the domains.

**Conclusion:**

The Arabic version of the IPQ-R for hypertensive patients is a consistent, valid, and reliable tool to be used by researchers or clinicians for assessing knowledge, beliefs, and attitudes of Arabic speaking patients with hypertension living in Saudi Arabia.

## Background

Hypertension is a major public health issue affecting over 1.28 billion adults (30–79 years) with a high mortality rate of 7.5 million deaths attributed to it every year worldwide (1, 2). The prevalence of hypertension in the Kingdom of Saudi Arabia (KSA) is 31.4% reflecting more than one fourth of the population of Saudi Arabia to be hypertensive, resulting in the leading cause of morbidity and mortality in the country (3). Unhealthy lifestyle, especially diet and exercise, play significant role in the progression of hypertension (4). Several antihypertensive drugs have been developed to treat hypertension. Moreover, traditional Chinese medicine injections (TCMIs) play important role in the management of hypertension especially when the patients have proteinuria (5). Given the public health burden it is necessary to determine the perception of hypertension among patients in the KSA.

Hypertension is defined as blood pressure ≥ 130/80 mmHg and ≥ 140/90 mmHg by American College of Cardiology/American Heart Association (ACC/AHA) and European Society of Cardiology/European Society of Hypertension (ESC/ESH), respectively (6). However, ESC/ESH definition is followed in Saudi Arabia as World Health Organization (WHO) still recommends this definition (7). 1996, Weinman developed and evaluated the Illness Perception Questionnaire (IPQ)—a novel method to assess the cognitive representations of illness (8). The IPQ comprised of five components: identity, cause, timeline, consequences, and control/cure (8). Moss-Morris et al. (9) revised the IPQ (IPQ-R) by adding subscales related to timeline perceptions, illness coherence, and emotional representations. The Illness Perception Questionnaire-Revised (IPQ-R) in English is a validated and reliable tool for assessing patients' perceptions of a variety of their illnesses, including musculoskeletal diseases (10), rheumatic diseases (11), mental health disorders (12), diabetes mellitus (13), and hypertension (14). The Chinese and Spanish versions of the IPQ-R have been found to be valid, reliable, and conceptually equivalent to the original English questionnaire in the setting of hypertension as well as other chronic diseases (15, 16). Studies have additionally employed other translated versions of the IPQ-R in Nepalese, Amharic, and Afaan Oromo languages to assess perceptions of hypertension, albeit these translated versions were not linguistically validated (17, 18). The IPQ-R has not been translated into any other languages in the context of hypertension.

Given that hypertension is a public health issue in Saudi Arabia, it may be pertinent to assess the illness perception among hypertensive patients in this country. Understanding patients' perceptions of their illness is the first step to improving patient care by understanding illness-related coping mechanisms and by developing individual interventions (8). The original IPQ-R is available in English while the official language of Saudi Arabia is Arabic. Therefore, to ensure that Arabic speaking patients perceive the meanings of the items included in the original IPQ-R the questionnaire should be translated into Arabic and then evaluated for its validity and reliability (19). Translation of the questionnaire requires best practices to ensure that the translated version is conceptually and linguistically equivalent to the original. The IPQ-R has been translated into Arabic in one study, to evaluate the illness perceptions of 316 patients with chronic illnesses in Algeria (19). However, this study did not evaluate the questionnaire in the context of hypertension (19).

To the best of the authors' knowledge there is no validated or reliable illness perception questionnaire or tool in the Arabic language to determine hypertensive patients' perceptions of their disease. We therefore aimed to estimate the validity and reliability of an Arabic version of the IPQ-R among patients with hypertension to determine their perception of the disease.

## Methods

### Aim, design, and setting of the study

This descriptive cross-sectional study aimed to translate the Revised Illness Perception Questionnaire (IPQ-R) into Arabic and validate the translated questionnaire among Arabic hypertensive patients from the outpatient departments of the Prince Sattam University Hospital and King Khalid Hospital (KKH) in Al-Kharj City in the Kingdom of Saudi Arabia.

### The translation

We employed an IPQ-R (Hypertension) which was modified from the IPQ-R, a frequently used instrument that assesses patients' perception of their illness. The IPQ-R (Hypertension) comprises three components: illness representation, causal attributions, and identity (20). All questions under illness representation and causal attribution were scored on a five-point Likert scale where 1 = strongly disagree and 5 = strongly agree. Illness representation consisted of 26 questions within 7 subscales pertaining to emotional representations (higher score indicating a stronger emotional impact of the condition), timeline (acute/chronic) (higher score indicating chronicity), Time-cyclical (higher score indicating higher variability), illness coherence (higher score indicating patients' increased awareness of their illness), personal control (higher score indicating patients' perceived efficacy in controlling their illness), treatment control (higher score indicating patients' belief that the treatment is Effective at controlling their illness), consequences (higher scores indicating more severe impact of the illness on patients' lives). Causal attribution components included 18 questions pertaining to 4 subscales psychological attributions, risk factor attributions, immune attributions, and chance attributions, with higher scores indicating a stronger belief that a factor is causally related to their disease. 19 symptoms associated with high blood pressure were included in the identity component. Responses to them were recorded binarily as *Yes* or *No*. Only those symptoms identified by patients as related to their hypertension counted toward the identity score.

A bilingual panel consisting of doctors who were proficient in English and Arabic was assembled to translate the IPQ-R into Arabic. Best practices were followed as set out by the Patient-Reported Outcome Consortium's instrument translation process (21). The panel members consisting of 1 general practitioner, 2 family medicine specialists, and 2 cardiologists were familiar with the management of hypertension. An external medical translator was on the panel to review and ascertain the accuracy of the translation and resolve differences in translation. A back translation of the Arabic IPQ-R was performed by another panel of doctors (consisting of 1 general practitioner, 1 family medicine specialist, and 1 cardiologist) and a medical translator who were blind to the original English questionnaire and the aims and objectives of the study to further ensure the equivalence of translation. The back translation was deemed satisfactorily similar to the English IPQ-R. Differences between the Arabic IPQ-R and the back-translated English IPQ-R were resolved after the original panel conferred with the medical translators.

### Patient enrolment

Non-probability convenient sampling was conducted at the general family medicine and cardiology outpatient departments of the Prince Sattam University Hospital and KKH in Al-Kharj City in the Kingdom of Saudi Arabia. Hundred patients were recruited from 1 August to 30 September 2021. Inclusion and exclusion criteria were followed. The inclusion criteria were adult patients of either sex older than 18 years of age, clinically diagnosed with primary hypertension for at least 6 months prior to the study, native Arabic by ethnicity, and fluent and literate in Arabic. The exclusion criteria were patients experiencing acute distressing symptoms or suffering complications of hypertension such as chest pain, hypertensive emergencies, or renal failure; diagnosed with secondary hypertension; diagnosed with other mental or physical comorbid conditions that could influence the perception of illness aside from hypertension such as diabetes, dyslipidemia, anxiety, or depression; patients unable to read the questionnaire such as those who cannot read Arabic or those with severe mental or visual impairment; and patients who did not give consent.

### Patient interviews

Cognitive debriefing interviews of ten participants were carried out to establish whether the participants interpreted the translated questions as intended and used the response scale appropriately. A standardized approach to cognitive debriefing was employed (22)—a trained investigator performed in-person semi structured interviews and used the think-aloud method. In the think-aloud method the participant completes the questionnaire by reading all items aloud and vocalizing their deciding processes and answers. The fact that the interviewer remains silent during the entire process mitigates interviewer bias. The participants provided their understanding of the questions in the questionnaire, the type of information needed to recall the answers, and the deciding processes undertaken to answer the questions appropriately. The participants were required to provide comments and general feedback about their experience in answering the questionnaire, e.g., if it was too long, if it caused significant mental or emotional burden, etc. The responses were manually transcribed by the interviewers, but were not included in the analysis of the validity of the questionnaire. They were used to revise the questionnaire as necessary.

### Ethical considerations

This study was approved by the Research Ethics Committee in Health and Science Disciplines of the Prince Sattam bin Abdulaziz University. Ethical approval number [REC-HSD-60-2021]. Informed consent was obtained from all the participants. Strict measures were observed to anonymize the personal identifiers of the participants to protect their privacy and confidentiality. Apart from the principal investigator who had access to the complete dataset all members of the research team were given access only to anonymized data.

### Data collection

Participants who met the inclusion and exclusion criteria were given the questionnaire for self-administration after their consultations. The participants completed the questionnaire in a dedicated room to ensure comfort and privacy. They were instructed to ask the investigator for clarifications if they had difficulties with understanding or answering the questions. The participants took an average of 10 min to complete the questionnaire. Demographic data such as age, sex, and socioeconomic status were recorded by the investigator.

### Statistical analysis

The SPSS (Statistical Package Social Science) software version 20 and Python programming language version 3.9 were used for statistical analysis. Descriptive statistics of the participants' characteristics were computed. Descriptive statistics of the IPQ-R domains and the frequency and percentages of the responses were also measured.

In order to measure reliability, test-retest analysis was performed on a random subsample of 10 patients to whom the questionnaire was administered twice with a gap of 6 weeks. Weighted Cohen's kappa with a linear weighting matrix was used for Likert scale items to compensate for the degree of disagreement. Simple Cohen's kappa was applied to binary items. Cronbach's alpha values were calculated for each domain separately and also for the overall Likert scale items for determination of internal consistency. The Kaiser-Meyer-Olkin (KMO) measure of sampling adequacy was applied to check the intercorrelation between each item of scale. KMO values > 0.40 were considered acceptable. Bartlett's Test of Sphericity was also applied to check if there was redundancy between the variables. A 95% confidence interval (CI) was calculated for coefficients.

## Results

### Characteristics of the participants

100 patients participated in this study and completed the IPQ-R. Fifty-seven patients (57%) were male and sixty-five (65%) were older than 40 years. Sixty nine percent of the population had a family history of hypertension and 49% were from the lower socioeconomic status group. Demographic details of the participants are presented in Table 1.

**Table 1:** Demographic profile of the patients.

**Demographic variables**		**Frequency**	**Percent (%)**
Age groups	40 or less	35	35%
	More than 40	65	65%
Gender	Male	57	57%
	Female	43	43%
Family history of Hypertension	Yes	69	69%
	No	31	31%
Socio-economic Status	Low	49	49%
	Middle	31	31%
	Upper	20	20%
**Total**		100	100%

### Disease identity domain

**Figure 1:**
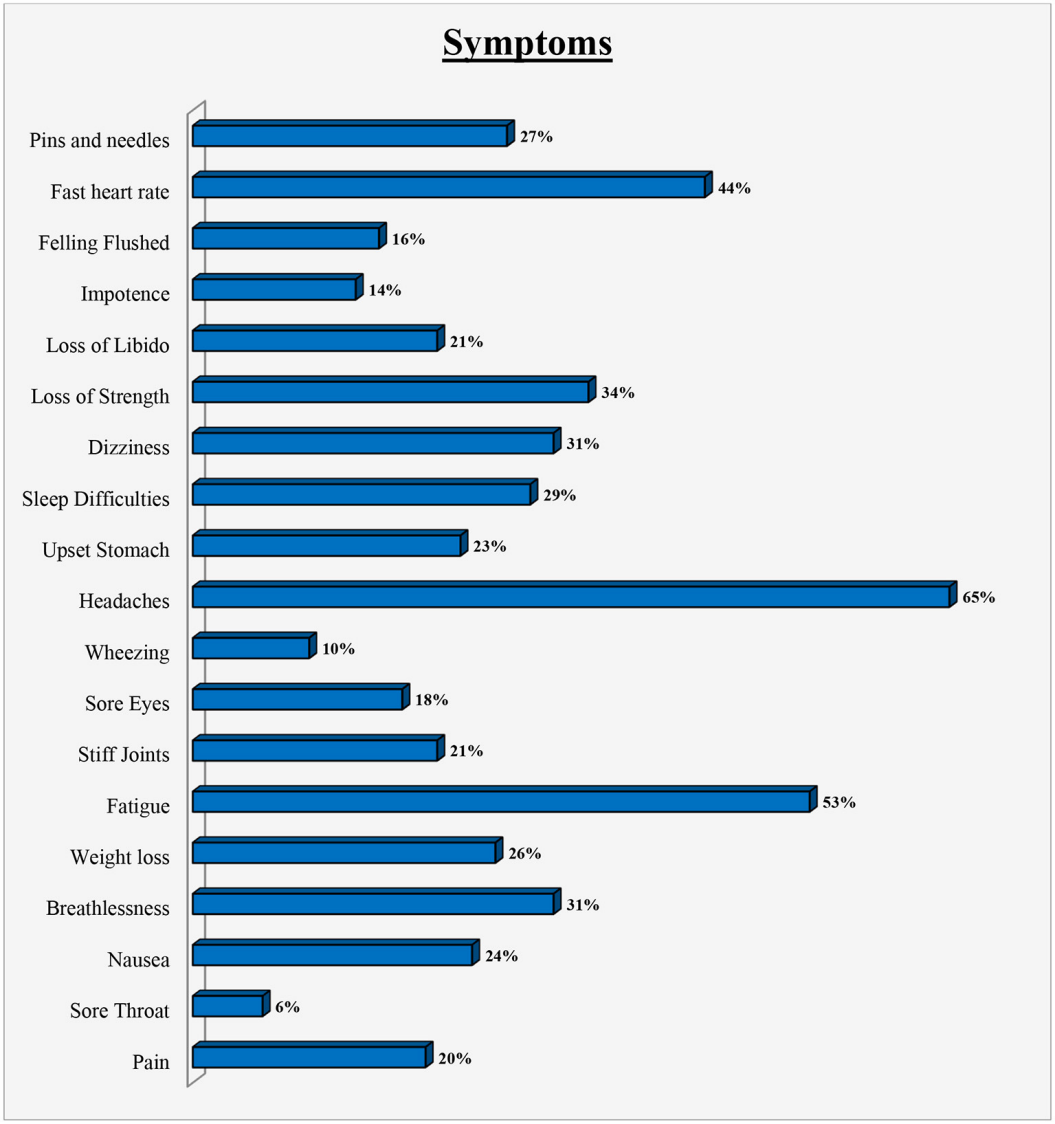
Symptoms experienced by patients.

The most frequently reported symptom of hypertension was headache in 65% of the participants, followed by fatigue (53%). Other common reported symptoms were fast heart rate (44%), loss of strength (34%), dizziness (31%), breathlessness (31%), and sleep difficulties as shown in Figure 1.

### Scores and reliability

The mean scores of the patient responses in total and by domain along with the Cronbach's alpha are presented in Table 2. Participants had the highest mean score (3.65, while SD = 0.66) in the domain of personal control which comprised 4 questions. Other domains in which the participants had high mean scores were illness coherence (mean = 3.41, SD = 1.09) and time-cyclical (mean = 3.41 SD = 1.09). The lowest scores were reported in the domains of causes (mean = 2.95, SD = 0.53) and consequences (mean = 2.73, SD = 0.71) which comprised 18 and 5 questions respectively. The domain of disease identity comprising 19 questions which were reported as a binary variable (no = 0 or yes = 1) had a mean of 0.27 and a standard deviation of 0.19.

**Table 2:** Descriptive Statistics of IPQ-R Domains (*n* = 100).

**IPQ–R Domains**	**Mean+/–SD**	**Items**	**Crobach's Alpha(α)**	**95% CI**
Disease Identity	0.27+/−0.19	19	0.78	0.74–0.84
Emotional Representations	3.08+/−0.76	5	0.72	0.62–0.79
Time	3.13+/−0.72	4	0.60	0.45–0.71
Time cyclical	3.41+/−0.56	4	0.44	0.24–0.60
Illness Coherence	3.41+/−1.09	1	—-	—-
Personal control	3.65+/−0.66	4	0.59	0.44–0.71
Treatment control	3.45+/−0.64	3	0.34	0.08–0.54
Consequences	2.73+/−0.71	5	0.54	0.38–0.67
Causes	2.95+/−0.53	18	0.77	0.70–0.83
Overall Domains	2.83+/−0.27	9	0.43	0.25–0.59
Overall items (Except Disease Identity)	3.19+/−0.31	8	0.42	0.23–0.58
All (Except Disease Identity)	3.10+/−0.33	44	0.76	0.69–0.82

IPQ–R, Illness Perception Questionnaire–Revised; SD, Standard deviation; α, Intraclass correlation; CI, Confidence interval.

**Figure 2:**
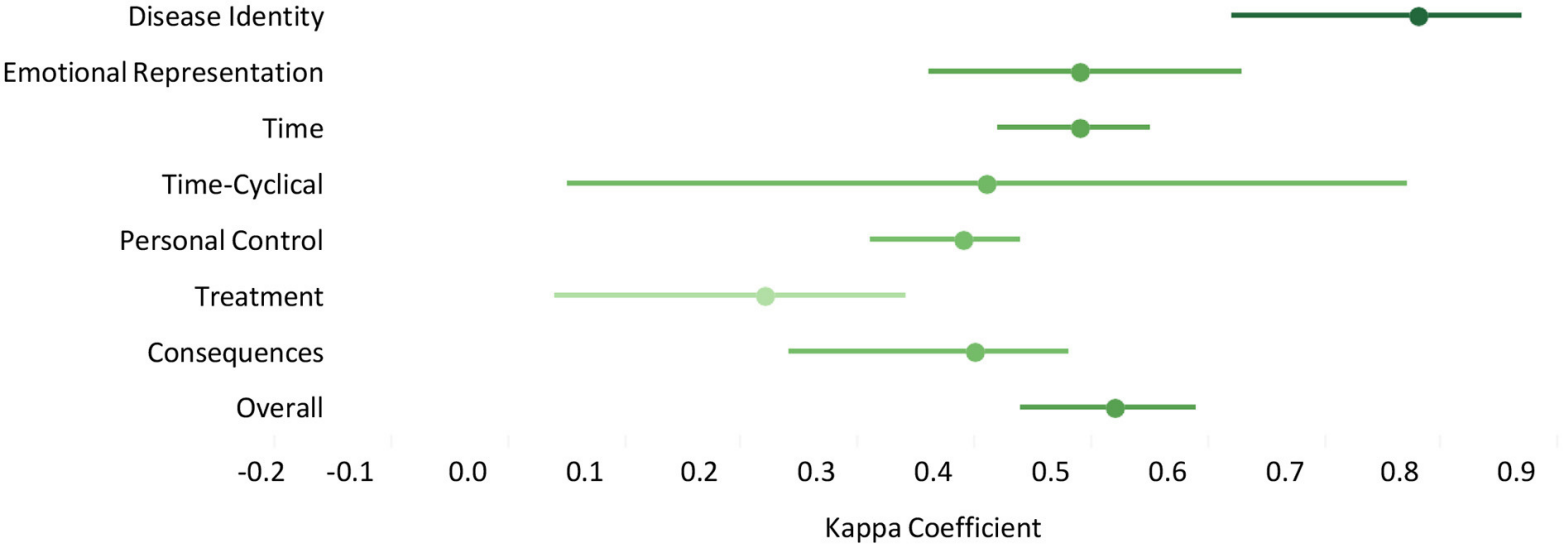
Kappa co-efficient with 95% confidence interval as determined by test-retest analysis.

Except for illness coherence which had only one question, the kappa coefficient was calculated for each domain, including disease identity (0.78; 95% CI: 0.62–0.87), emotional representation (0.49; 95% CI: 0.36–0.63), time (0.49; 95% CI: 0.42–0.55), time-cyclical (0.41; 95% CI: 0.05–0.77), personal control (0.39; 95% CI: 0.31–0.44), treatment (0.22; 95% CI: 0.04–0.34), consequences (0.40; 95% CI: 0.24–0.48), and causes (0.39; 95% CI: 0.24–0.51). The kappa coefficient of the questionnaire was 0.52 (95% CI: 0.44–0.59) overall. Kappa coefficients with 95% confidence interval as determined by the test-retest analysis are depicted in Figure 2.

As described in Table 2, the internal consistency of the questionnaire was determined within each domain and for the whole questionnaire using Cronbach's alpha. Within the domains the Cronbach's alpha levels ranged from 0.34 in the treatment control domain to 0.78 in the disease identity domain. The internal consistency of the whole questionnaire excluding the domain of disease identity was 0.76 indicating satisfactory internal consistency. Given that the disease identity domain was measured on a dichotomous (yes/no) scale compared to the 5-point Likert scale response for the illness representation and the casual attribution domains, it was omitted when computing the Cronbach's alpha for the whole questionnaire.

### Intercorrelations of the domains of the IPQ–R

There were weak to moderate positive linear correlations (r = 0.003–0.561) between the domains of the IPQ–R as shown in Table 3 suggesting a reasonable discriminant validity among the domains. The domain of emotional representation has a weak positive correlation to time (r = 0.258) and a moderate positive correlation to consequences (r = 0.478) (*p* < 0.01). The time-cyclical domain has a weak positive correlation to personal control (r = 0.265, *p* < 0.01) and treatment control (r = 0.315, *p* < 0.01). Personal control has a moderate positive correlation to treatment control (r = 0.561, *p* < 0.01) and a weak positive correlation to consequences (r = −0.240, *p* < 0.01) and illness coherence (r = 0.421, *p* < 0.01). Treatment control and illness coherence have a weak positive correlation (r = 0.260, *p* < 0.01) as do consequences and causes (r = 0.425, *p* < 0.01).

**Table 3:** Inter–Correlation between subscales of the IPQ–R (*n* = 100).

**IPQ–R Domains**	**Disease Identity**	**Emotional Representations**	**Time**	**Time cyclical**	**Personal control**	**Treatment control**	**Consequences**	**Illness Coherence**	**Causes**
Disease Identity	1								
Emotional Representations	0.180	1							
Time	0.075	0.258**	1						
Time cyclical	0.229*	0.032	0.078	1					
Personal control	0.122	−0.106	−0.052	0.265**	1				
Treatment control	0.065	−0.083	−0.129	0.315**	0.561**	1			
Consequences	−0.061	0.478**	0.256*	−0.135	−0.240**	−0.242	1		
Illness Coherence	0.094	0.003	0.066	0.038	0.421**	0.260**	−0.098	1	
Causes	0.052	0.234*	0.036	−0.008	−0.046	0.023	0.425**	−0.123	1

*P < 0.05, **P < 0.10. IPQ–R, Illness Perception Questionnaire–Revised.

### Variable collinearity

The Kaiser-Meyer-Olkin (KMO) test and Bartlett's Test of Sphericity were carried out to test variable collinearity. We obtained a KMO measure of 0.549 and a significance level of < 0.001 for the Bartlett's statistic indicating substantial correlation in the data.

## Discussion

The primary purpose of this cross-sectional study was to produce a linguistically equivalent and culturally acceptable translation of the IPQ-R into the Arabic language for patients with hypertension. Cronbach's alpha, a measure of reliability, showed satisfactory internal consistency among the domains of disease identity, emotional representation, and causes in the Arabic translated IPQ-R. In research, internal consistency measures the extent to which the observed results show trustworthy answers to the questionnaire (23). The satisfactory internal consistency of some items in the study shows the appropriateness and meaningfulness of the process used to translate the questionnaire into Arabic and conduct cognitive debriefing. Test-retest analysis showed the disease identity domain to have the highest kappa values, shown to be in the substantial range of agreement with the overall questionnaire kappa values in the moderate range of agreement for reliability. The study reported weak to moderate positive linear correlations between IPQ–R domains, representing the importance of individual domains.

Validity and reliability are critical elements to evaluate a questionnaire. Cronbach's alpha is frequently used in medical research when multiple items are employed to construct a concept (24). In this regard, application of Cronbach's alpha reported significant internal consistency in terms of main domains of the translated IPQ-R in the present study. To further strengthen the reliability, test-retest analysis was applied which showed highest values for the domain of “disease identity”. Hence, the reliable statistical tools used in the study are important to validate the translated IPQ-R.

Aberkane (19) translated the IPQ-R into Arabic and adapted it for Algerian patients with chronic illnesses. They reported Cronbach's alpha coefficient consistently higher than 0.45 (quite reliable) in terms of internal consistency of the Arabic version which they created. Although Cronbach's coefficient of 0.45 in Aberkane's study (19) did not fall within the range of the “very reliable” category, it opened doors to further research on the assessment of validity and reliability of the Arabic version of the IPQ-R for patients with chronic illnesses. However, this study was not designed for the Arabic hypertensive patients, but for patients with chronic illnesses in general. In this context it is reasonable to adduce that a single questionnaire for assessing many diseases is less sensitive as it may not detect minute but clinically significant perceptions specific to the disease (25). Chinese translations of the IPQ-R have been employed on hypertensive patients and judged to have conceptual and linguistic equivalence to the English counterpart as determined by satisfactory Cronbach's alpha values indicating good internal consistency, and by confirmatory factor analysis that provided evidence of satisfactory factorial validity, convergent validity, and discriminant validity (15). Spanish translations of the IPQ-R have been linguistically validated among patients with hypertension and other chronic conditions (16).

Variables collinearity represents the extent to which two or more independent variables included in the study can predict the values of each other (26). In the present study the data represented significant variable collinearity indicating that the items in the questionnaire consistently measured patients' perceptions of hypertension. However, this study revealed a weak to moderate positive linear correlation between the domains, showing that the elements or domains of the translated version of the IPQ-R have discriminant validity.

The importance of this study is that this is the first study which translated the IPQ-R into the standard Arabic language specifically for hypertensive patients and reported satisfactory internal consistency among three of nine IPQ-R domains and variable collinearity. The use of standard Arabic furthermore allows for potential use of this questionnaire in other Arabic speaking countries.

### Limitations

Illness perceptions of hypertension may change with the duration, severity, and complications of hypertension. Since our study included limited patients, further study is required to validate the questionnaire in patients with severe hypertension or those who have experienced complications related to hypertension. Studies at multicenter and multinational levels are also required to ascertain the generalizability of the translated version in different cultural contexts and other Arabic speaking countries. A sample of 100 is smaller than other similar studies (27, 28). The relatively small sample size limited performing the principal component analysis and factor analysis of the Arabic translated questionnaire.

In conclusion, given that Cronbach's alpha values for most of the subscales in the Arabic translated version of the IPQ-R for hypertensive patients were unsatisfactory (< 0.7), this Arabic translation of the IPQ-R did not show sufficient internal consistency to adequately ascertain the illness perceptions of hypertensive patients living in Saudi Arabia. However, the overall questionnaire kappa as ascertained by test-retest analysis was in the moderate range of agreement for instrument reliability. Future studies can employ questionnaires other than the IPQ-R which are validated for hypertension or employ alternative Arabic translations of the IPQ-R to ascertain illness perceptions of Arabic speaking hypertensive patients.

## Data availability statement

Data are available upon request from the authors.

## Ethics statement

The Research Ethics Committee in Health and Science Disciplines in the Deanship of scientific research, Prince Sattam bin Abdulaziz University with IRB (REC-HSD-60-2021) approved this study. Written informed consent was obtained from the study participants after explaining the aim of the study to each of them.

## Author contributions

SA-G, YT, AB, and AAA participated in the study design. SA-G wrote the first draft of the manuscript. AMA and NA collected and processed the samples. YT, AB, and AAA performed the statistical analyses. All authors read and approved the final manuscript.

## Funding

This publication was supported by the Deanship of Scientific Research at Prince Sattam bin Abdulaziz University, Al Kharj, Saudi Arabia.

## Conflict of interest

The authors declare that the research was conducted in the absence of any commercial or financial relationships that could be construed as a potential conflict of interest.

## Publisher's note

All claims expressed in this article are solely those of the authors and do not necessarily represent those of their affiliated organizations, or those of the publisher, the editors and the reviewers. Any product that may be evaluated in this article, or claim that may be made by its manufacturer, is not guaranteed or endorsed by the publisher.
